# Key tips for teaching in the clinical setting

**DOI:** 10.1186/s12909-020-02283-2

**Published:** 2020-12-03

**Authors:** Annette Burgess, Christie van Diggele, Chris Roberts, Craig Mellis

**Affiliations:** 1 The University of Sydney, Faculty of Medicine and Health, Sydney Medical School - Education Office, The University of Sydney, Edward Ford Building A27, Sydney, NSW 2006 Australia; 2 The University of Sydney, Faculty of Medicine and Health, Sydney Health Professional Education Research Network, The University of Sydney, Sydney, Australia; 3 The University of Sydney, Faculty of Medicine and Health, The University of Sydney, Sydney, Australia; 4 The University of Sydney, Faculty of Medicine and Health, Sydney Medical School – Central Clinical School, The University of Sydney, Sydney, Australia

**Keywords:** Clinical teaching, Clinical tutorials, Clinical reasoning, Bedside teaching, Role modelling, Near-peer teaching, Peer-peer teaching

## Abstract

Teaching with real patients in the clinical setting lies at the heart of health professional education, providing an essential component to clinical training. This is true of all the health disciplines – particularly medicine, nursing, dentistry, physiotherapy, and dietetics. Clinical tutorials orientate students to the culture and social aspects of the healthcare environment, and shape their professional values as they prepare for practice. These patient-based tutorials introduce students to the clinical environment in a supervised and structured manner, providing opportunities to participate in communication skills, history taking, physical examination, clinical reasoning, diagnosis and management. It is only through participation that new practices are learnt, and progressively, new tasks are undertaken. The aim of this paper is to provide health professional students and early career health professionals involved in peer and near peer teaching, with an overview of approaches and key tips for teaching in the clinical setting. Although there are many competencies developed by students in the clinical setting, our tips for teaching focus on the domains of medical knowledge, interpersonal and communication skills, and professionalism.

## Background

Although simulation is increasingly used in health professional education, the long-held tradition of teaching with the involvement of real patients, remains invaluable. Teaching within the clinical setting, such as bedside and out-patient clinic, lies at the heart of healthcare education, providing a vital component to clinical training. These tutorials orientate students to the culture and social aspects of the clinical environment, and shape students’ professional values as they prepare for practice [1]. They offer students meaningful opportunities to participate in clinical activities, practicing and developing their communication skills, history taking and physical examination competence. However, students’ learning in the clinical environment is largely dependent upon the affective, pedagogic and organisational support afforded to them [2–6].

Peer and near peer tutoring are well accepted as sources of support within healthcare curricula, particularly in the clinical setting, where participation involves a process of socialisation [3, 6]. Clinical tutors act as socialising agents, demonstrating the expected culture and professional values of their respective health professions, and their organisation. That is, clinical tutors, whether peer-to-peer, or clinician to student, demonstrate key components of the ‘hidden curriculum’ [7]. The aim of this paper is to provide health professional students and early career health professionals involved in peer and near peer teaching, with an overview of approaches and key tips for teaching in the clinical setting. Although there are many competencies developed by students in the clinical setting, our tips for teaching focus on the domains of medical knowledge, interpersonal and communication skills, and professionalism.

### Tips for teaching with patients

Bedside and out-patient (office-based) teaching remains a primary teaching modality in the clinical setting, where many aspects of clinical practice can be taught and modelled [8]. A holistic approach in the diagnostic process and patient care is provided in bedside teaching, where history taking, physical examination skills and professional attitude are combined [8]. As a general rule, patients enjoy being included in the teaching process. Essentially, teaching with patients permits three key learning domains to be integrated with teaching [9]:

1. Clinical (knowledge and skills).
2. Professionalism (teamwork, ethical considerations).
3. Communication (with staff and patients).

Healthcare students find interactions with some patients to be challenging, particularly if the patient is hostile, angry, uncooperative, disinterested, overly talkative, or experiencing chronic pain [10]. When teaching with a patient there a number of important considerations:

- Incorporate interactions as “key teaching moments”, with opportunities for tutors to help students develop competence in communication skills [11]
- Ensure patient involvement in education, and patient centredness [12]
- Respect the comfort and rights of patients, whether in the presence of the patient, or otherwise [13, 14]
- Always obtain the patient’s consent
- Ensure the patient is prepared for their role through clear communication
- Allow the patient to ask questions and give feedback
- Use appropriate language that the patient can understand
- Have a specific purpose/objective for the teaching session
- Limit the time the student spends with the patient by identifying the tasks and timeframe for the student
- Provide feedback (particularly negative feedback) to the student away from the patient
- Be aware that it may not be appropriate to discuss some patient conditions in front of a group

Part of the role of clinical tutors is to facilitate the process of socialisation into the healthcare profession, creating a sense of identity relating to the students’ current and future roles in healthcare [15–20]. Tutors are entrusted with responsibilities to foster students’ learning, helping to develop students’ attitudes, values and professional competencies. Three core characteristics of a positive role model include [15–20]:

1. Clinical attributes
2. Personal qualities
3. Teaching skills

Displays of humanistic behaviours, encompassing empathy, respect and compassion for patients are of the utmost importance to students [16–18, 21–26]. Undesirable behaviours by clinical tutors include tutor-centred patient interactions; the humiliation of students; and negative remarks about colleagues [18, 24]. Table 1 summarises positive and negative attributes of clinical teachers as role models, identified within health professional education [18, 27].

**Table 1:** Positive and negative attributes of clinical teachers as role models

***Clinical Attributes***	
**Positive**	**Negative**
Good knowledge of medicine, able to articulate history taking skills	Inability to impart knowledge at the student level
Empathy, respect and compassion for patients	Lack of empathy, respect or compassion for the patients
Recognises own limitations	Lack of awareness of own limitations
***Personal Qualities***	
**Positive**	**Negative**
Clearly prepares for the tutorials	Lack of enthusiasm for teaching and the subject
Respectful interactions amongst all hospital staff	Lack of respect for members of staff
***Teaching Skills***	
**Positive**	**Negative**
Provision of good patient interaction	Lack of patient interaction
Positive learning environment, and a good rapport with students	Humiliation of students
Structured tutorials, clear expectations	Poorly structured tutorials
Understanding of the curriculum and assessment requirements	A poor understanding of the curriculum and assessment requirements
Observation of student performance, and provision of immediate, meaningful feedback	Lack of direct observation, and lack of meaningful feedback

### Tips for planning teaching

**Fig. 1:**
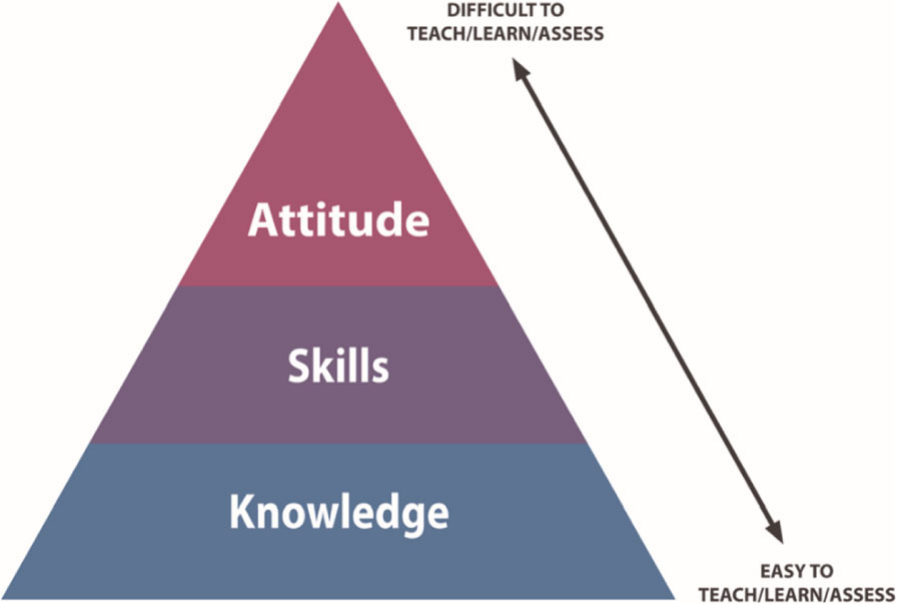
Ease and difficulty of teaching, learning and assessing knowledge, skills and attitude

Ensure that your teaching session is well planned and any assessments are aligned with the learning outcomes and content [28]. Important considerations include:

- Use of a framework, such as ‘Outcomes, Activity, Summary’ (OAS) (Table 2).
- How will your teaching session link to previous (for example, lectures) and future learning activities (for example, formative assessments)?
- How will you incorporate the teaching of knowledge, skills and attitudes (Fig. 1) into your session, with ‘knowledge’ being more easily imparted and assessed than ‘skills’ and ‘attitudes’ [29]
- What will be the role of the teacher, the learner, and the patient, and how will each individual contribute to the session?
- Are there aspects of the teaching environment that require consideration? These might include how the students will be placed around the bedside, or in the outpatient room; patient confidentiality; and where briefing and debriefing will take place (booking of a tutorial room may be required).

**Table 2:** The ‘OAS’ method for lesson planning and teaching

**Outcomes**	
Consider the background knowledge of the learners	
Establish learning outcomes for the teaching session	
Plan the set up of the environment (seating, etc)	
**Activity**	
Plan how you will engage learners in the teaching activity	
Encourage students to actively contribute to the session	
Involve all students actively in the session, for example, take turns in taking a history, or share parts of the task	
**Summary**	
Summarise the knowledge and skills covered	
Ensure the session finishes on time	
Reflect on the teaching session and seek feedback

### Tips for teaching strategies

**Fig. 2:**
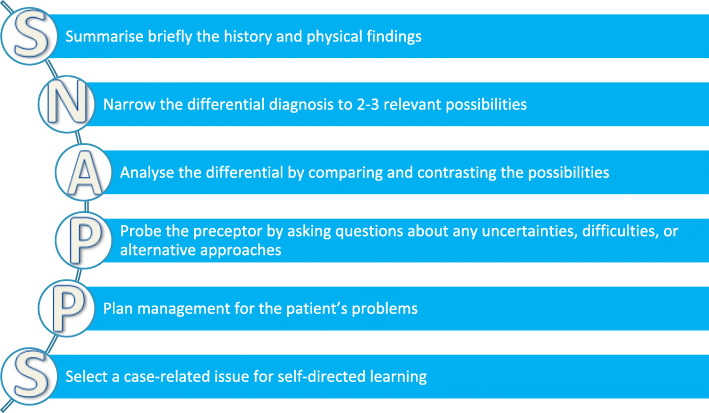
The SNAPPS model [30]

Apply an appropriate teaching strategy, such as SNAPPS (Fig. 2), developed as a mnemonic for a learner-centred teaching model for case presentations in the outpatient/office setting [30]. The benefits of the SNAPPS format include:

- encourages a structured and brief presentation by the student
- engages the learner to explore, and express their own knowledge gaps (that is, the student “probes” the tutor about their uncertainties)
- compared to more traditional tutor interactions, learners are more actively involved, and ask more questions
- enables tutors to address each learners’ specific, individual needs [30].

#### Clinical reasoning

Do your best to promote the learners’ clinical reasoning [31, 32] - the cognitive process underlying diagnosis and management of a patient’s presenting problem. The process involves:

- The collection of data
- Diagnostic reasoning
- Therapeutic reasoning
- Planning intervention and recommendations

**Fig. 3:**
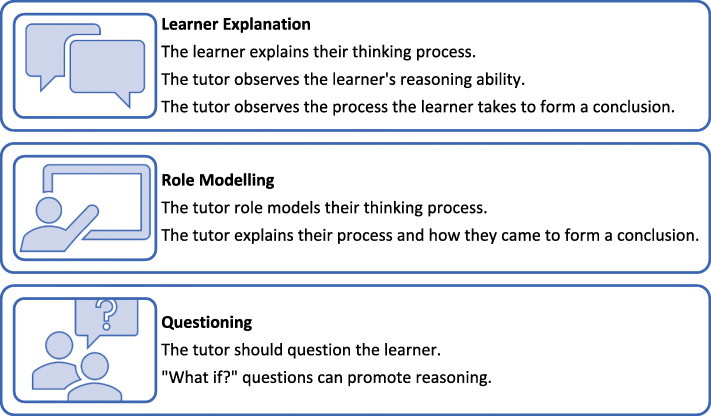
Three tips for promoting clinical reasoning

The process of integrating and applying knowledge to patient care is a complex, difficult skill for students to acquire [32–34]. In particular, it is difficult for students to navigate patient information during patient interactions. There are three main ways to promote clinical reasoning [24, 31, 35] (Fig. 3):

1. *Learner explanation:* The learner explains their thinking process, allowing the tutor to observe the learner’s reasoning ability, and the process they take to form a conclusion.
2. *Role modelling*: The tutor role models their own thinking process, explaining their reasoning (‘thinking aloud’).
3. *Questioning the learner*: The tutor uses questioning to promote reasoning, “what if?” questions are asked.

A suggested framework for teaching clinical reasoning [34], which was adapted from Peyton’s model [36], is shown in Table 3. Teaching clinical reasoning also provides an excellent opportunity for tutors to reflect on their own clinical reasoning skills. The benefits of reflection may include the avoidance of assumptions, reduction of unnecessary investigations, and improvement in time to diagnosis [34].

**Table 3:** Clinical reasoning framework (adapted from Linn et al., 2012) [34]

**1. Demonstration and deconstruction** The clinical tutor demonstrates a patient interaction at normal speed. The tutor then clearly explains their thinking and reasoning to the student.	
**2. Comprehension** The student actively tracks the consultation, outlining the clinical reasoning process being demonstrated. The teacher pauses to allow the student to explain what they understand is happening.	
**3. Performance** The student performs the history taking and physical examination, and suggests investigations. The student explains their reasoning to the tutor as they proceed, and the tutor offers clarity throughout the process.	

### Tips for assessment strategies

Assessment provides a key driving force for learning. It reinforces the information and skills learned, provides the learner with information on their areas of strength and weakness, and provides the teacher with information on areas that may need to be re-taught [14, 37]. In order for the assessment activity to be worthwhile students need clear outcomes, an indication of their performance against these outcomes and guidelines on how to improve [37–39].

The utility or usefulness of an assessment has been defined as a product of its reliability, validity, cost-effectiveness, acceptability, educational impact and feasibility [40–43]. Factors to consider when selecting and creating an appropriate assessment for students include [14]:

1. *Reliability:* refers to the reproducibility of the scores obtained from an assessment if repeated under similar circumstances [41].
2. *Validity:* refers to whether an instrument actually does measure what it is purposed to [41]. Evidence of the validity supports the use of the results of an assessment for a particular purpose [43].
3. *Feasibility:* refers to whether the assessment is practical, realistic, and sensible, given the circumstances and context [43]. Constraints on an ideal assessment include availability of examiners, the time of academics to develop the material, administrative resources to implement assessments, faculty training requirements and analysis of assessment [42].
4. *Acceptability:* refers to the extent to which the assessment, the assessment process and results are considered credible by the stakeholders [43].
5. *Educational impact:* refers to the educational effects of assessment on both the learner and the curriculum; including unexpected impacts [40].
6. *The cost of an assessment:* refers to lengthy tests having major resource implications, both in terms of testing time as well as in terms of cost to produce these tests. This impacts decisions on the effective use of resources in a sustainable way [40].

#### Formative assessment methods in the clinical setting

Formative assessment offers a powerful tool to inform the learner of their progress at a particular point in time [14]. In recent years, formative assessments have been reshaped and formalised to suit the clinical setting [44]. These improvements have evolved from a previously loosely planned clinical immersion, to a curriculum-based experience linked to achievement of pre-determined outcomes. A number of well described formative assessment methods (Table 4), suitable for providing feedback based on direct observation in the clinical setting, have been developed in recent years [45]. In order to gain a well-rounded understanding of a learner’s performance and ability, increasingly, formative assessment takes place over multiple occasions. This allows the assessor to have multiple opportunities to observe and monitor communication skills, procedural skills, levels of professionalism, clinical skills and general competence [38, 39, 46].

**Table 4:** Examples of formative assessment methods

Name	Description
Direct observation of procedural skills (DOPS)	Usually a checklist approach to measuring procedural skills
Mini Clinical Evaluation Exercise (Mini-CEX)	A focused component of a clinical encounter (eg. a targeted history, or a focused physical examination, or a communication skill).
Case-based Discussion (CbD)	A trainee discusses a case with a supervisor, the case notes may also provide triggers to guide discussion.
Formative (practise) long case clinical examination	Learner will see a patient and then afterwards discuss the patient’s condition and management in depth with the examiners.
Formative (practise) Objective Structured Clinical Examination (OSCE)	Designed for rating clinical skill performance and competence. eg. communication, physical examination, procedural skills.
Multi-source feedback (MSF)	Designed for rating professional behaviour. Ratings may come from peers, supervisors, patients. They include the benefit of aggregating multiple perspectives of performance.

### Tips for provision of effective feedback

Feedback to students forms a crucial part of the learning process, and should always be included within clinical tutorials. Tips for provision of effective feedback are outlined in Table 5. A meta–analysis exploring the effect of feedback on clinical performance found that the provision of feedback had a positive impact in over 75% of the included studies [47]. Effective and regular feedback has the potential to promote self-reflection, reinforce good practice; and directs the learner to practice towards the required outcome [37]. Feedback has the greatest impact on students’ behaviour when it is based on direct observation, and is immediate [37, 48]. Existing feedback frameworks, such as Pendleton’s model [49] is learner-centred, and offers the learner the opportunity to evaluate their own practice. First, the tutor asks the learner what they think they did well, then describes areas that were done well; then the tutor asks the learner how they could improve, and then suggests to the learner how they can improve. Whatever model of feedback is chosen, feedback from the tutor should be honest, descriptive and specific.

**Table 5:** Tips for provision of effective feedback

*Tips for provision of effective feedback*	
• Make a direct observation	
• First ask the learner for a ‘self-assessment’	
• Be constructive	
• Provide specific detail on what went well, and what needs improvement	
• Limit the feedback to two or three specific areas for improvement	
• Provide a detailed strategy on how to achieve improvement	
• Check the learner clearly understands what needs improvement, and how to work towards improvement	
• Plan another observation and feedback session	
• Document the session	

## Conclusion

Teaching in the clinical setting, and particularly, bedside teaching is viewed by patients, students and tutors as an invaluable teaching method. To optimise learning and maximise student engagement, learning activities in the clinical environment should be planned, structured, and aligned with the curriculum, and assessment [50]. Since students learn largely through observing and imitating their tutors, role modelling plays a critical role in influencing students’ learning and behaviour. Role modelling by clinicians, and by senior students, assists in the development of healthcare students’ professional competencies, values, and attitudes. Feedback plays a crucial role in the learning process. By observing, and providing students with accurate feedback, the gap between actual and desired performance is narrowed.

### Take-home message

**Table:** 

• Always ensure the rights of patients are respected when teaching and learning activities take place in the clinical setting. • Successful teaching activities are well planned, with a structured format. • Structured teaching methods, such as “SNAPPS”, help to format the session. • Direct observation and provision of feedback is essential to student learning.	

## Data Availability

Not applicable.

## Abbreviations

OAS: Outcomes, Activities, Summary
SNAPPS: Summarise, Narrow, Analyse, Probe, Plan, Select
DOPS: Direct observation of procedural skills
Mini-CEX: Mini Clinical Evaluation Exercise
CbD: Case-based Discussion
OSCE: Objective Structured Clinical Examination
MSF: Multi-source feedback
